# Multifocal Signal Modulation Therapy by Celecoxib: A Strategy for Managing Castration-Resistant Prostate Cancer

**DOI:** 10.3390/ijms20236091

**Published:** 2019-12-03

**Authors:** Roberto Benelli, Paola Barboro, Delfina Costa, Simonetta Astigiano, Ottavia Barbieri, Matteo Capaia, Alessandro Poggi, Nicoletta Ferrari

**Affiliations:** 1 Immunology, IRCCS Ospedale Policlinico San Martino, L.go R. Benzi 10, 16132 Genova, Italy; simonetta.astigiano@hsanmartino.it; 2 Academic Unit of Medical Oncology, IRCCS Ospedale Policlinico San Martino, L.go R. Benzi 10, 16132 Genova, Italy; paola.barboro@hsanmartino.it; 3 Molecular Oncology & Angiogenesis, IRCCS Ospedale Policlinico San Martino, L.go R. Benzi 10, 16132 Genova, Italy; delfina.costa@hsanmartino.it (D.C.); alessandro.poggi@hsanmartino.it (A.P.); nicoletta.ferrari@hsanmartino.it (N.F.); 4 Department of Experimental Medicine (DIMES), University of Genova, Via L.B. Alberti, 16132 Genova, Italy; ottavia.barbieri@unige.it; 5 Department of Internal Medicine and Medical Specialties (DIMI), School of Medicine, University of Genoa, Via Balbi 5, 16126 Genova, Italy; matteo.capaia@hsanmartino.it

**Keywords:** celecoxib, signaling, castration-resistant prostate cancer, ErbB family, inflammation, apoptosis

## Abstract

Background: Prostate cancer (PCa) is a significant health concern throughout the world. Standard therapy for advanced disease consists of anti-androgens, however, almost all prostate tumors become castration resistant (CRPC). Progression from androgen-sensitive PCa to CRPC is promoted by inflammatory signaling through cyclooxygenase-2 (COX-2) expression and ErbB family receptors/AKT activation, compensating androgen receptor inactivity. Methods: Making use of CRPC cell lines, we investigated the effects of the anti-inflammatory drug celecoxib. Biochemical data obtained using immunoblotting, enzyme-linked immunosorbent assay (ELISA), invasion, and xenografts were further integrated by bioinformatic analyses. Results: Celecoxib reduced cell growth and induced apoptosis through AKT blockade, cleavage of poly (ADP-ribose) polymerase-1 (PARP-1), and proteasomal degradation of the anti-apoptotic protein Mcl-1. Epidermal growth factor receptor (EGFR), ErbB2, and ErbB3 degradation, and heterogeneous nuclear ribonucleoprotein K (hnRNP K) downregulation, further amplified the inhibition of androgen signaling. Celecoxib reduced the invasive phenotype of CRPC cells by modulating NF-κB activity and reduced tumor growth in mice xenografts when administered in association with the anti-EGFR receptor antibody cetuximab. Bioinformatic analyses on human prostate cancer datasets support the relevance of these pathways in PCa progression. Conclusions: Signaling nodes at the intersection of pathways implicated in PCa progression are simultaneously modulated by celecoxib treatment. In combination therapies with cetuximab, celecoxib could represent a novel therapeutic strategy to curb signal transduction during CRPC progression.

## 1. Introduction

Prostate cancer (PCa) is the most frequently diagnosed neoplasia in men in developed countries and the second leading cause of cancer-related death in Europe and USA [1] with 1.3 million new cases diagnosed in 2018. According to the statistics, incidence and mortality percentages are rapidly increasing in the Chinese aging society too [2]. The causes of PCa have not been fully clarified and, over heritable factors, immigration studies highlight the involvement of environmental exposures. Despite advances in screening and early detection, the morbidity for PCa remains high and a large percentage of men continue to present advanced or metastatic disease. As androgens regulate prostate cancer growth, androgen deprivation therapy (ADT) remains the standard first line approach for metastatic hormone-sensitive PCa (mHSPC). The duration of ADT response is approximately 18–24 months, then most patients progress to the more aggressive castration-resistant prostate cancer (CRPC) although castrate levels of testosterone are ≤20 ng/mL. Gene expression studies provided some explanations about CRPC development. Among them alterations and/or constitutive activation of androgen receptor (AR)-dependent pathways, re-expression of androgen-responsive genes downregulated by ADT [3], and androgen receptor (AR) gene amplification have been detected in circulating CRPC cells [4,5].

The widespread presence of chronic inflammation in pathological specimens from prostate tissue has sustained its link with PCa development [6]. Prostatic inflammation generates free radicals that upregulate cyclooxygenase enzymes thus increasing the production of eicosanoids and prostaglandins, recognized as prostate cell proliferation inducers [6,7]. In human benign prostatic hyperplasia (BPH) tissues, cyclooxygenase-2 (COX-2) inhibition produces a significant increase in prostate cell apoptosis [8]. Accordingly, we demonstrated that nonsteroidal anti-inflammatory drugs (NSAIDs) could act as beneficial chemopreventive agents in hormone-sensitive PCa cell lines [9]. In the present study, we treated two androgen-resistant LNCaP sublines (PDB and MDB) [10], that recapitulate some phenotypic features of PCa evolution to CRPC, with the COX-2 inhibitor celecoxib. In particular, PDB cells mimic the clinical condition in which cancer cells are partially exposed to androgens due to inadequate therapy or cancer cells producing androgens in the local microenvironment. Conversely, MDB cells mimic the clinical condition in which cancer cells survive despite a completely hormone-deprived microenvironment. In vitro, most of the signaling pathways responsible for CRPC progression were inhibited by celecoxib. Tumor cell growth inhibition by celecoxib was further confirmed in vivo in a mouse model of CRPC. By providing a new drug combination, CRPC could—at least—become a chronic disease. There are no curative medications currently in sight.

## 2. Results

### 2.1. Effects of Celecoxib on the Growth and Apoptosis of the CRPC Cell Lines MDB and PDB

**Figure 1:**
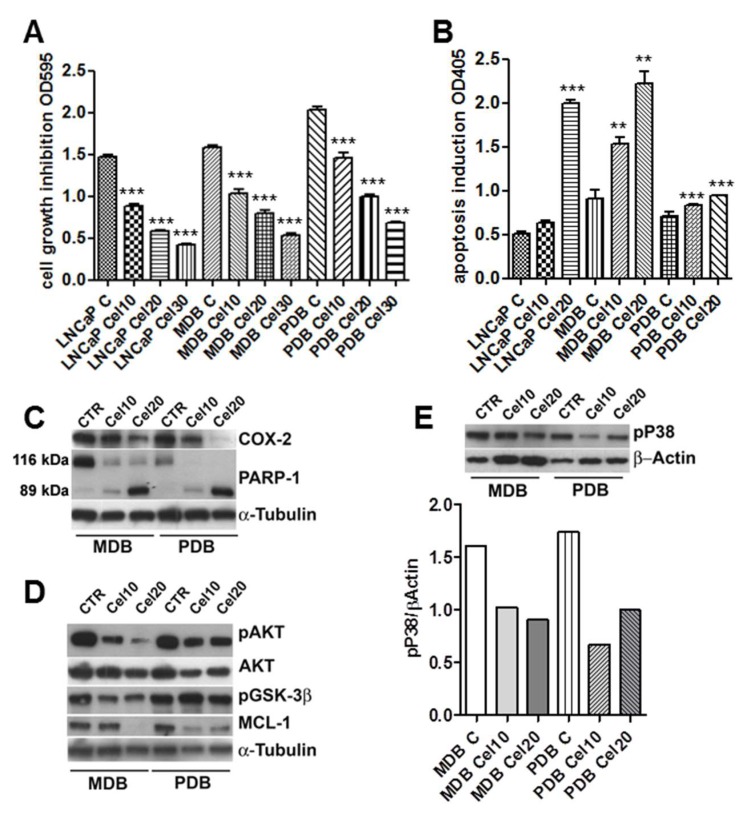
Celecoxib regulates castration-resistant prostate cancer (CRPC) cell growth and apoptosis modulating pAKT and pP38 activities. (**A**) Crystal violet cell viability in CRPC cell lines MDB and PDB compared to androgen responding LNCaP cells. Cells were grown 72 h with 10, 20, and 30 μM celecoxib in a medium supplemented with 5% FBS. Values represent one out of three independent experiments performed on 10 samples. Mean ± SD are reported. (**B**) Apoptosis induction by celecoxib in MDB and PDB resistant cells compared to that in LNCaP cells. Cells were treated with celecoxib and apoptosis was evaluated after 48 h. Values are representative of one out of three experiments carried out in triplicate. Mean ± SD are reported. * *p* < 0.05, ** *p* < 0.01, *** *p* < 0.001. (**C**) Effect of celecoxib on cyclooxygenase-2 (COX-2) expression and poly (ADP-ribose) polymerase-1 (PARP-1) cleavage in resistant cells. Western blotting analyses showing native (116 kDa) and cleaved (89 kDa) PARP-1 protein bands demonstrate that 24 h treatment with celecoxib dose-dependently induced PARP-1 cleavage. (**D**,**E**) Cells were treated with celecoxib for 48 h and lysates (4 μg/lane) were analyzed for pAKT, AKT, pGSK3β, and Mcl-1 (**D**) and pP38 (**E**) by SDS-PAGE and Western blotting with specific antibodies. α-tubulin and β-actin were used as loading controls. Densitometric quantification of pP38 decrease by celecoxib is also reported (E, lower panel).

To investigate the effect of celecoxib-induced cell viability, MDB and PDB cells were treated with various concentrations of the drug. Celecoxib decreased the cell viability in a dose-dependent manner. After 72 h, 30 μM celecoxib strongly reduced MDB and PDB cell viability to about 35% (Figure 1A). Further studies were thus conducted in the presence of 10 and 20 μM celecoxib.

Normal prostate epithelial cells are insensitive to celecoxib-induced apoptosis [9,11] suggesting a correlation between COX-2 expression and apoptosis sensitivity. COX-2 expression in LNCaP cells was in fact higher than that in normal prostate epithelial cells [11]. COX-2 expression in the parental LNCaP and resistant MDB and PBD cell lines was quite similar (data not shown) and celecoxib treatment decreased its expression in a dose-dependent manner in resistant cells (Figure 1C). In order to determine whether the cytotoxic effect of celecoxib was due to apoptosis, MDB and PDB cells were treated with increasing concentrations of the drug. As in the parental LNCaP cell line, apoptosis significantly increased in MDB and PBD cells following administration of 10 and 20 μM celecoxib (Figure 1B). We previously demonstrated that prolonged bicalutamide (BIC) exposure induced genome instability in MDB and PDB cell lines driving activation of the DNA repair pathway, as confirmed by the upregulation of the DNA repair enzyme PARP-1 [10]. PARP-1 is a death substrate cleaved and inactivated by downstream caspases in response to growth factor removal or exposure to chemotherapeutic agents. To determine whether PARP-1 is cleaved during celecoxib-induced apoptosis, MDB and PDB cells were treated with 10 and 20 μM celecoxib for 48 h and monitored for PARP-1 cleavage with an antibody specifically recognizing the 89 kDa fragment of cleaved PARP-1 and the uncleaved 116 kDa protein. As shown in Figure 1C, celecoxib dose-dependently increases the 89 kDa cleavage product and decreases the 116 kDa uncleaved PARP-1. No 89 kDa fragments of PARP-1 were detected in untreated cells, providing evidence for apoptosis induction upon celecoxib treatment.

### 2.2. AKT Phosphorylation Is Inhibited by Celecoxib

We know, from our previous studies and in agreement with findings on tissues from CRPC patients [10,12,13], that androgen-resistant cell survival is supported by the activation of two signaling pathways: AKT and p38MAPK (P38). We thus tested whether celecoxib could attenuate the activity of the anti-apoptotic kinase AKT. MDB and PBD cells were exposed to 10 and 20 μM celecoxib for 48 h and examined by Western blot for AKT activation. Figure 1D shows the impact of celecoxib treatment on phospho-AKT levels, the inhibition was particularly relevant in the MDB cell line. Under identical conditions pP38 activity was also modulated in MDB and PDB cells (Figure 1E).

In the absence of pAKT, the AKT target protein glycogen synthase 3β (GSK3β) is dephosphorylated and triggers the phosphorylation of the anti-apoptotic Mcl-1 that in turn, after ubiquitination, undergoes proteasomal degradation [14]. Cellular extracts from treated MDB and PDB cells showed that celecoxib modulates GSK3β phosphorylation in MDB cells (Figure 1D). Mcl-1 protein levels decreased proportionally in celecoxib-treated cells (Figure 1D).

### 2.3. Celecoxib Attenuates AR Expression and Function in Resistant Cells through ErbB Receptor Inhibition and *Epidermal Growth Factor* (EGF) and Amphiregulin (AREG) Induction

**Figure 2:**
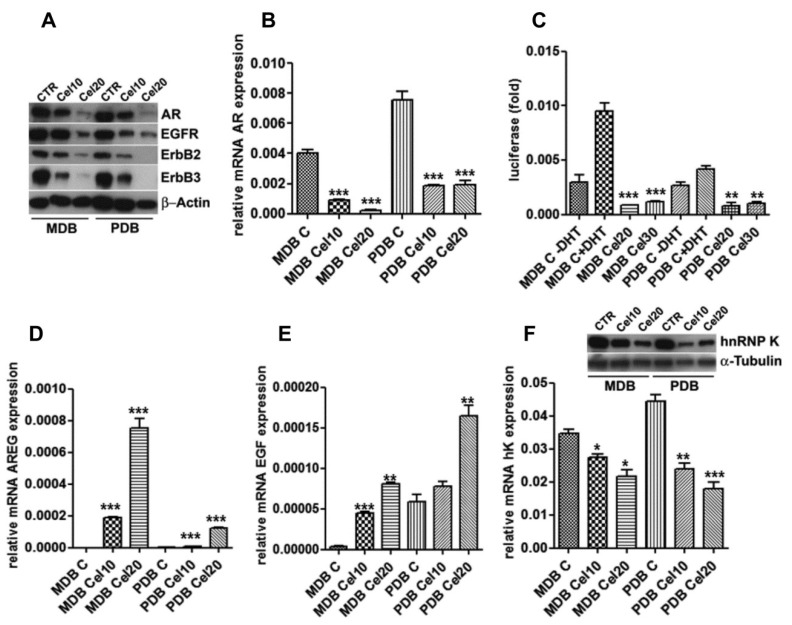
Celecoxib modulation of androgen receptor (AR) expression associates with ErbB receptors, heterogeneous nuclear ribonucleoprotein K (hnRNP K) downregulation and epidermal growth factor (EGF) and amphiregulin (AREG) induction. (**A**) AR, epidermal growth factor receptor (EGFR), ErbB2, and ErbB3 levels are downregulated by celecoxib after 48 h. β-actin was used as a loading control. (**B**) Real-time RT-PCR on mRNAs from cells cultured as in panel A indicates AR decrease also at the transcriptional level. (**C**) AR reporter gene activity. Cells transfected with the dual luciferase AR reporter gene, in the presence or absence of 10 nM 5-α-dihydrotestosterone (DHT), were exposed to increasing amounts of celecoxib. Luciferase activity is normalized to renilla activity. Mean ± SD of three experiments conducted in triplicate are reported. (**D**) AREG and (**E**) EGF mRNA levels obtained by quantitative real-time RT-PCR in MDB and PDB cells treated with celecoxib for 24 h. Target transcripts were corrected by the corresponding 18S transcript levels. Mean ± standard error (SEM) of one out of three experiments conducted in triplicate are shown. (**F**) HnRNP K transcriptional control by celecoxib. HnRNP K mRNA and protein levels were analyzed by real-time RT-PCR and Western blotting with specific primers and antibodies utilizing the material as in panel A. α-tubulin was used as a loading control. * *p* < 0.05, ** *p* < 0.01, *** *p* < 0.001.

We previously reported the ability of celecoxib to modulate the EGFR-AR signaling in androgen-responsive PCa cells, yielding a rationale for its inclusion in chemopreventive strategies [9]. In MDB and PDB, celecoxib reduced AR at mRNA and protein levels (Figure 2A,B) in a dose-dependent manner.

Although the transcriptional activity of AR in resistant cells was almost nil [10], when AR function was measured using a luciferase reporter gene, celecoxib further impaired both basal and 5-α-dihydrotestosterone (DHT)-induced AR activation (Figure 2C).

AR controls EGFR and ErbB2 expression in prostate cancer cells and in turn EGFR and ErbB2 concur to PCa progression activating AR signaling in hormone-poor conditions [15]. Celecoxib decreased EGFR, ErbB2, and ErbB3 protein levels (Figure 2A), with the ErbB2 and ErbB3 decrease particularly evident at 20 μM. In LNCaP responding cells, celecoxib caused EGF and AREG induction, EGFR and ErbB2 activation and degradation, and finally inhibition of the androgenic signaling [9]. AREG and EGF basal mRNA levels were almost undetectable in both MDB and PDB resistant cells and significantly increased, in a dose-dependent manner, in celecoxib-treated cells (Figure 2D,E).

### 2.4. Celecoxib Regulates AR through hnRNP K

ErbB receptors may regulate the expression of the RNA binding protein hnRNP K [16], whose binding to the long 3′ UTR of AR and COX-2 can regulate their mRNA stability and thus protein expression [17,18]. Since a functional link between AR and hnRNP K has been reported [19,20], we tested whether celecoxib could modulate hnRNP K and then AR expression. Twenty-four hours of celecoxib treatment were sufficient to reduce hnRNP K mRNA and protein levels (Figure 2F).

### 2.5. Downregulation of Constitutive NF-κB Activity by Celecoxib Reduces Resistant Cell Invasion

**Figure 3:**
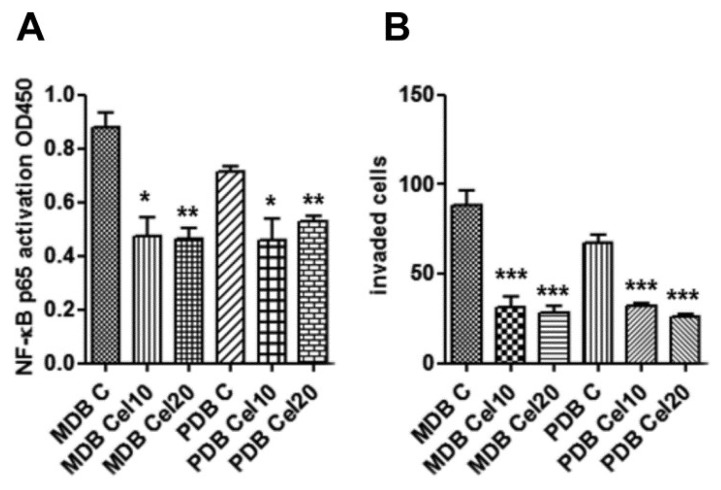
Downregulation of constitutive NF-κB activity by celecoxib associates with reduced MDB and PDB invasiveness. (**A**) Enzyme-linked immunosorbent assays (ELISAs) show that 24 h exposure to celecoxib inhibited constitutively active NF-κB in resistant cells. (**B**) Celecoxib inhibition on the capability of MDB and PDB cell lines to invade Matrigel in response to human fibroblast-conditioned medium. Means ± SD of two experiments performed in triplicate are reported: * *p* < 0.05, ** *p* < 0.01, *** *p* < 0.001.

NF-κB signaling is involved in carcinogenesis, cancer progression, metastasis, and drug resistance [21,22]. In breast cancer, the NF-κB pathway has been shown to be activated downstream of ErbB2, and ErbB2-induced signaling pathways include MAPK and PI3K/AKT [23]. As celecoxib impaired AKT, P38, and ErbB2 activities, we investigated celecoxib effects on NF-κB. Enzyme-linked immunosorbent assay (ELISA) analyses indicate that a 24 h treatment inhibited the amount of active NF-κB in both MDB and PDB resistant cells (Figure 3A). As NF-κB modulates the expression of factors controlling tumor cell motility, invasiveness, and/or metastasis [24], we assessed the invasiveness of the two resistant cell lines in response to human fibroblast-conditioned medium. Celecoxib significantly prevented invasion of both MDB and PDB cells (Figure 3B). These results indicate that, in our model system, the constitutive activation of NF-κB could drive CRPC development, as recently described [25].

### 2.6. Effects of Celecoxib and Cetuximab Alone or in Combination on the Growth of MDB Xenografts in Immunodeficient Mice

Based on the in vitro findings reported above, we investigated whether celecoxib had similar effects in vivo. Compared to the parental LNCaP cells, unable to induce tumors in NOD/SCID male mice, the acquisition of an aggressive phenotype by MDB and PDB cells has been reported [10].

**Figure 4:**
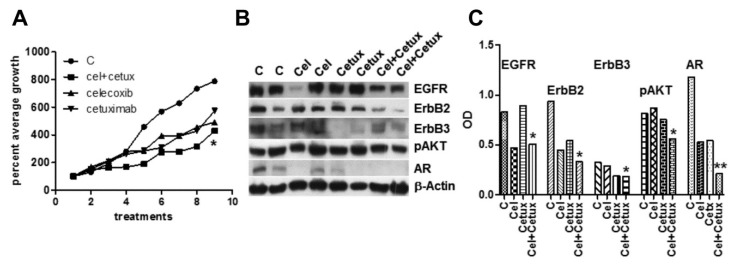
Effects of celecoxib and/or cetuximab on the growth of androgen-independent MDB xenograft. Male NOD SCID mice were injected s.c. with MDB cells in 50% Matrigel (5.0 × 10^6^ cells/200 μL). (**A**) MDB tumor bearing mice were injected peri-tumor with celecoxib (20 mg/kg) or i.p. with cetuximab (200 μL) or both compounds. Treatments and measurements were repeated three times per week. Tumor sizes were expressed as percent of initial tumor size. (**B**) Western blot analyses of tumor extracts from control and treated animals confirmed decreased signaling described in in vitro experiments and particularly evident in samples from combined treatments. (**C**) Densitometric analysis of bands reported in panel (**B**) normalized by β-actin values. * *p* < 0.05, ** *p* < 0.01.

Celecoxib is able to amplify EGFR activation in primary colorectal cancer-associated fibroblasts triggering sustained Erk1-2 and AKT signaling [26]. A strong fibroblast activation was also detected in vivo, under chronic celecoxib treatment [27]. In our in vivo model of xenografts, mouse fibroblasts could be further activated by celecoxib-induced secretion of EGF and AREG by epithelial tumor cells and by a stress environment characteristic of castration-resistant cells [10,28]. Thus, we tested in vivo celecoxib also in association with the EGFR inhibitor cetuximab. Mice injected with MDB cells were separately given celecoxib, or cetuximab, or both. The growth curves, represented as average percent of the first experimental point (Figure 4A), indicate a visible, but not significant slowdown induced by single compounds, which became significant (*p* < 0.05) in the association.

Combined treatments also significantly modulated the main signaling pathways as proved by Western blotting analyses of lysates from tumor samples (Figure 4B,C). Examination of hematoxylin and eosin (H&E)-stained tumor sections from the four groups, revealed that the peritumor injection of celecoxib did not induce tissue damage or fibrotic reaction compared to the other conditions (Figure S1).

### 2.7. Biological and Clinical Relevance of the Celecoxib-Controlled Gene Set (CGS) in Prostate Cancer: A Bioinformatics Analysis

**Figure 5:**
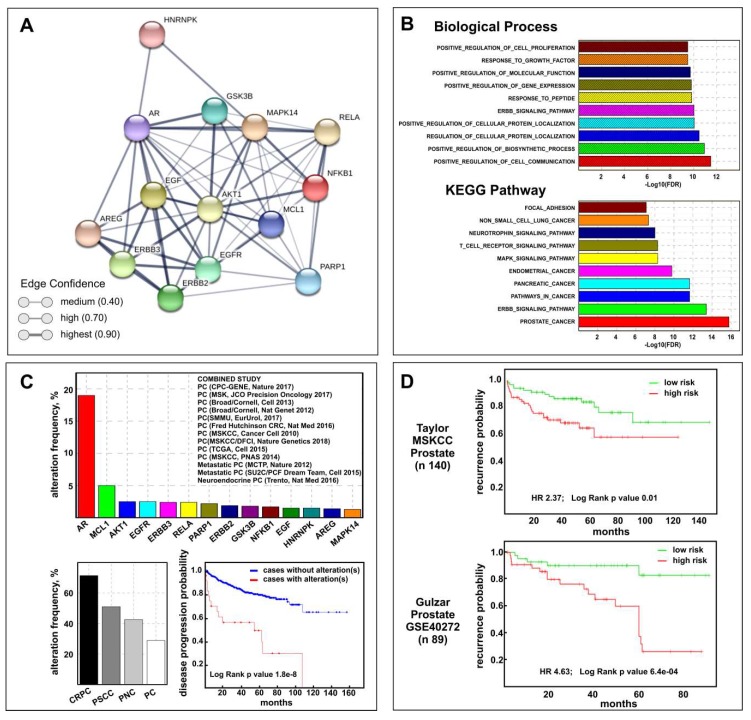
Biological and clinical relevance of the celecoxib-controlled gene set (CGS) in prostate cancer. (**A**) STRING visualization of celecoxib-controlled protein network. Line thickness edge indicates the strength of data support (confidence). (**B**) Gene set enrichment analysis (GSEA) histograms for CGS enrichment analysis of PCa samples (TGCA PRAD dataset). The −Log10 (FDR-corrected *p* value) is plotted for the top ten gene ontology-biological process (GO-BP) (**top**) and KEGG pathways (**bottom**). (**C**) CGS cancer genomic analysis results by cBioPortal: genetic alteration frequency of CGS genes (excluding COX-2) and the 13 prostate datasets used are shown (**top**); CGS genetic alterations frequency distribution in different subtype according to cBioPortal terminology: castration-resistant prostate cancer (CRPC), prostate small cell carcinoma (PSCC), prostate neuroendocrine carcinoma (PNC), and prostate adenocarcinoma (PC) (**bottom left**); Disease progression-free survival Kaplan–Meier graph of patients with or without CGS genetic alterations (**bottom right**). (**D**) Kaplan–Meier plots showing recurrence in Taylor MSKCC (**top**) and Gulzar (**bottom**) datasets, depending on differential expression of each gene across the CGS (excluding COX-2), as reported on SurvExpress Website.

The above results suggest that celecoxib may be useful for therapy of PCa progression. To validate this assumption, we defined the CGS composed by the 15 genes (*AKT1, AR, EGFR, ERBB2, ERBB3, GSK3B, MAPK14 (P38), MCL1, PARP1, COX2, AREG, EGF, HNRNPK, NFKB1, RELA*) modulated by celecoxib treatment in our in vitro CRPC models. Using the Drug Signature Database (DSigDB) browser, providing gene expression changes induced by over 12,000 approved drugs, we verified that 80% of CGS genes were really celecoxib target genes (Table S1). Interestingly, performing network analysis by STRING software, a network among all CGS proteins was generated (Figure 5A) demonstrating a clear relationship among them.

To understand the biological effects and mechanisms associated with the CGS, we performed gene ontology-biological process (GO-BP) and pathway enrichment analysis by CSEA. The top ten GO-BPs mainly concern mechanisms involved in response to external stimuli while prostate cancer pathways were found among the top ten KEGG pathways (Figure 5B). The combined STRING analysis of the CGS here identified and the 172 genes and/or proteins previously described as differentially expressed in MDB and PDB cells during castration resistance development [10] resulted in a high number of interactions and a highly significant protein–protein interaction (PPI) enrichment *p*-value (Figure S2A) mainly determined by *AR* and *AKT1* (Figure S2B). With this premises, we verified the CGS clinical relevance by performing bioinformatics analyses on human prostate cancer genomic and transcriptomic datasets using respectively the cBioPortal and the SurvExpress Web resources. Cancer genomic analysis using 13 multidimensional prostate cancer data sets (Figure 5C) showed that significantly higher genetic alterations were observed for *AR* (19%) and *MCL1* (5%) genes among the CGS. Of note, the prostate cancer subtype with the highest percent of CGS genetic perturbation was CRPC (71.4%) while only 28.9% was observed in PCa. Using the Kaplan–Meier method we found that genetic and mRNA expression alterations of the CGS are associated respectively with disease progression (Figure 5C) and recurrence (Figure 5D)-free survival for PCa patients.

## 3. Discussion

Although the new therapies recently approved by the United States Food and Drug Administration for advanced PCa (Hematology/Oncology Cancer Approvals and Safety Notifications), in 2019, about 32,000 men have been predicted to die from PCa in the United States [1]. Today, almost all patients dying from prostate cancer have CRPC.

During PCa progression, cell heterogeneity is caused by genomic rearrangements and mutations targeting specific biological processes and signaling pathways [29,30]. Phenotypic plasticity and clonal heterogeneity [10] induced by anticancer therapies may further amplify ADT defeat through environmental factors driving “non-genetic” heterogeneities [31,32].

Several recent Phase III trials (GETUG-AFU 15, CHAARTED, and STAMPEDE) have demonstrated that docetaxel chemotherapy and ADT for mHSPC synergistically leads to improvements in overall survival compared to ADT alone in hormone-naïve patients. These data suggest that the initiation of ADT induces susceptibilities in PCa cells that make them sensible to synergistic treatments [33,34,35].

To simulate PCa patients receiving medical or surgical castration combined with ADT, we developed two androgen-resistant LNCaP sublines by treating the cells for a long time with BIC in presence (PDB) and absence (MDB) of DHT. Molecular and functional analyses of these resistant sublines identified phenotypic features of PCa evolution to CRPC.

**Figure 6:**
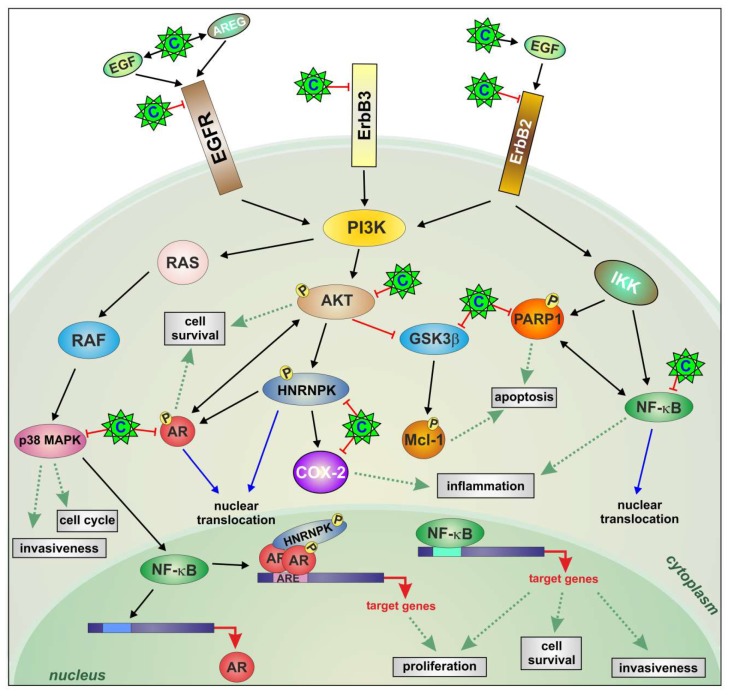
Celecoxib modifies the relationship among inflammation, ErbB receptors, and COX-2 in CRPC. A constitutively activated signaling composed of ErbB family receptors/AKT/P38/AR/NF-κB is directly inactivated by celecoxib in CRPC cells. Celecoxib reduced cell growth and induced apoptosis with AKT blockade, PARP-1 cleavage, and proteasomal degradation of the anti-apoptotic protein Mcl-1. Through EGF and AREG induction, celecoxib caused further EGFR and ErbB2 activation and consequent degradation associated with the inhibition of androgenic signaling amplified by ErbB3 and hnRNP K downregulation, implicated in castration-resistance and AR transcription and translation control respectively. Positive and negative regulations are depicted in black and red (arrows and T-bars, respectively). Green dotted arrows indicate cellular processes. Celecoxib (C).

We here demonstrate that a constitutive activation of ErbB family receptors controlling AKT/AR/GSK3β/P38/NF-κB and hnRNP K has emerged in PCa cells during the progression to CRPC. All these molecules, representing signaling nodes at the intersection of pathways implicated in cancer progression, are simultaneously modulated by celecoxib treatment (Figure 6).

The CGS we defined does really contain celecoxib target genes detected and modulated in PCa patients (Table S1). Interestingly, the analysis of hierarchical relationships among the top ten GO-BP highly enriched in deregulated genes and proteins during CRPC progression [10] and in CGS showed common GO terms (Figure S2C) suggesting that celecoxib could really regulate CRPC features. Notable, Kaplan–Meier estimates confirm that genetic and mRNA expression alterations of the CGS may be associated respectively with disease progression (Figure 5C) and recurrence probability (Figure 5D).

A lot of literature reports that CRPC arises from few tumor cells surviving first-line ADT therapy and no longer responding to the therapy [36]. Therefore, disruption of cell survival mechanisms during ADT seems a promising strategy through which CRPC could be prevented. From our combined STRING analysis (Figure S2A,B) AKT and AR emerged as key proteins in cell survival. The ErbB family has been implicated in PCa initiation and progression to CRPC [37,38,39] as it can turn on the PI3K/AKT pathway and regulate AR transcriptional activity in a ligand-independent manner [40,41]. Thus, it may be favorable to inhibit the ErbB receptors directly. We here provide evidence that celecoxib, switching-off ErbB receptors, reduces AKT and AR activities attenuating the major anti-apoptotic AKT pathway [11] followed by GSK3β activation and degradation of the anti-apoptotic proteins Mcl-1 and PARP-1 (Figure 6). Downstream PI3K, P38 signaling inhibition modulates cell survival, migration, invasion [42], and NF-κB activation [43], known to contribute to prostate cancer progression and castration resistance [44] through the regulation of AR activity [45]. Indeed, combination treatments with AR and NF-κB inhibitors have shown promising results [46]. We show that celecoxib can counteract NF-κB activity through the control of PI3K downstream signaling and AR activity (Figure 6). Further, decreased prostaglandin levels by COX-2 downregulation can further contrast the inflammatory condition and prostate cell proliferation [6,7] (Figure 6).

In PCa, increased expression levels and altered phosphorylation of hnRNP K [47] modify its nuclear interaction with AR promoting the migration into the cytoplasm, where it cannot correctly control mRNA translation [19]. In androgen-dependent PCa and CRPC in vitro models, serine-phosphorylated hnRNP K isoforms are associated with different AR activities and a specific hnRNP K–AR signature indicates progression toward CRPC [48]. Celecoxib modulation of hnRNP K could provide additional mechanisms controlling AR transcriptional activity (Figure 6).

Previously reported data indicated that selective COX-2 inhibitors provided an overall 68% cancer risk reduction [49]. In one study, 78 men were randomly assigned to either celecoxib (400 mg/twice daily) or the placebo group. Twenty percent of men in the placebo group and 40% of men in the celecoxib group had posttreatment more than 200% doubling time of baseline PSA with no new metastases (*p* < 0.08) [50]. These results were curbed by observations of a possible risk to the cardiovascular system. However, “a meta-analysis of independent estimates from 72 studies provided no evidence that the selective COX-2 inhibitor celecoxib influences the relative risk of cardiovascular disease” [51]. Our data provide new premises for the revaluation of celecoxib in the clinical management of prostate cancer. In combination therapies celecoxib could represent a therapeutic strategy to weaken AR/ErbB crosstalk and resistance signaling in prostate cancer progression.

## 4. Materials and Methods

### 4.1. Cell Lines and Reagents

The BIC-resistant cell lines PDB and MDB, obtained in our laboratory, were cultured as described [10]. They were authenticated using STR profiling and all experiments were performed with mycoplasma-free cells. Celecoxib was from LKT Laboratories (St. Paul, MN, USA) while clinical grade cetuximab (5 mg/mL) was from the IRCCS Ospedale Policlinico San Martino Pharmacy. Celecoxib stock solutions (300 mM in DMSO) were maintained at −20 °C and freshly diluted in each experiment. The final concentration of DMSO in culture was 0.02%. Cell viability was evaluated by the trypan blue dye-exclusion technique. All drug treatments were performed in 5% FBS.

### 4.2. Cell Proliferation, Apoptosis, Invasion, and ELISA Assays

In vitro cell proliferation analysis was performed in 96-well plates with 2800 cells/well grown in complete medium or treated as described. The number of viable cells was evaluated by the crystal violet assay after 72 h.

To measure cytoplasmic histone-associated DNA fragments induced by celecoxib, a commercial kit was employed (Cell Death Detection, Roche, Basel, Swiss). Thirty thousand cells per well, 24-well plates, were grown in the presence of increasing concentrations of the drug for 48 h.

Cell invasion assays were carried out in cell-Matrigel chambers (BD Bio Coat, Bedford, MA, USA) following manufacturer’s instructions.

NF-κB activity was evaluated by enzyme linked immunosorbent assay (ELISA) using the Trans-AM NF-κB p65 Transcription Factor Assay Kit (Active Motif, Rixensart, Belgium), following the manufacturer’s instructions.

### 4.3. Reporter Assay

The Cignal androgen receptor dual luciferase reporter (Qiagen, Hilden, Germany) was transfected into the cells by Lipofectamine 2000 (Thermo, Carlsbad, CA, USA) according to manufacturer’s instructions. To activate transcription, DHT, 10 nM was added 15 h after transfection in the presence/absence of celecoxib and luciferase activity was assayed in triplicates after 48 h using the Dual-Luciferase reporter assay kit (Promega, Madison, WI, USA). Renilla luciferase was cotransfected in all reporter assays to control for transcription. Luciferase values represent ratios of luciferase/renilla.

### 4.4. RNA Isolation and Real-Time RT-PCR

Total RNA was isolated from the cells by the RNeasy Mini kit (Qiagen). mRNA expression was quantified by real-time reverse transcription-PCR with specific primers reported in Table S2. cDNA amplification and expression values were obtained as described [52].

### 4.5. Protein Extraction and Western Blot

Proteins were obtained from control and treated cells as reported in figure legends. Cells were lysed in protease inhibitors containing RIPA buffer and protein concentration quantified with the DC Protein Assay kit (Bio-Rad, Hercules, CA, USA). Cell lysates (4 μg/lane) were separated by SDS-PAGE, transferred to PVDF and probed at 4 °C overnight with the specific primary antibodies listed in Table S3. Protein bands were detected by chemiluminescent HRP substrate (Immobilon Western, Millipore, Burlington, MA, USA) and Hyper film-ECL (GE-healthcare, Chicago, IL, USA).

### 4.6. In Vivo Studies

Animals were housed and maintained by the Animal Care Facility at the IRCCS Ospedale Policlinico San Martino of Genova, according to national and European regulations (D.L. 4/3/14 no. 26; 86/609/EEC Directive). Experiments were approved by the internal Ethic Committee and received the approval by the Italian Ministry of Health (project no. 4/2016 to Nicoletta Ferrari). Five- six week-old immunocompromised NOD/SCID male mice were obtained from the breeding program of the Policlinico Animal Care Facility and inoculated subcutaneously with 5 × 10^6^ MDB cells, in a volume of 200 μL containing Matrigel (BD) and 10 μM BIC. To follow tumor growth all animals were regularly palpated three times per week and nodules were measured with a caliper. Volumes were calculated by the formula d_1_ × d_2_ × d_3_/2 where d represents the diameter. Based on the growth of the first tumor, all animals were subsequently treated when the nodule reached a volume between 80 and 90 mm^3^. Twenty-four tumor-bearing mice were randomly divided into four groups: the control group, the celecoxib group, the cetuximab group, and the celecoxib plus cetuximab group. Celecoxib suspension diluted in 40% PEG 6000 and saline, was administered at a dose of 20 mg/kg in the peri-tumor area, while 200 μL cetuximab (5 mg/mL) were given i.p. Control animals received solvents alone. Treatments were carried out three times per week.

Animals were euthanized by CO_2_ asphyxiation at the established endpoint or when showing any sign of suffering. At autopsy, tumors were excised and a portion fixed in 10% buffered formalin and paraffin-embedded for histopathological and immunohistochemical studies. A second portion of tumor was snap frozen and stored at −80 °C for molecular analyses.

### 4.7. Immunohistochemistry (IHC)

Tissue sections were cut at 4 µm and processed using the automated protocol of the Leica Bond Rx Automated Stainer (Leica Biosystems, Wetzlar, Germany). H&E images were captured using the 20× objective of the AperioAT2 scanner (Leica Biosystems).

### 4.8. Bioinformatics Analyses

The CGS bioinformatics analyses were performed using web-accessible programs: theDSigDB; http://tanlab.ucdenver.edu/dsigdb), the Search Tool for the Retrieval of Interacting Genes/Proteins (STRING v11.0; https://string-db.org/), the Gene Set Enrichment Analysis—Broad Institute (GSEA; http://www.broadinstitute.org/gsea/) and the QuickGO web service (https://www.ebi.ac.uk/QuickGO/). Mutation events and copy number alterations of CGS were associated with clinical data using the cBioPortal query Web interface (http://cbioportal.org) with a combined study across 13 prostate cancer datasets for a total of 3460 patients and 3676 samples. To evaluate recurrence-free survival, the mRNA expression of CGS genes were analyzed using Taylor MSKCC and Gulzar (GSE40272) datasets on SurvExpress Website (http://bioinformatica.mty.itesm.mx:8080/Biomatec/SurvivaX.jsp).

### 4.9. Statistical Analysis

Statistical differences were evaluated with an unpaired Student’s *t*-test for two groups’ comparison and two-way analysis of variance (ANOVA) for multiple-group comparison. Data are expressed as mean ± standard error (SEM), and *p* values < 0.05 were considered statistically significant.

## Supplementary Materials

Supplementary materials can be found at https://www.mdpi.com/1422-0067/20/23/6091/s1. The following are available online: Figure S1: Comparison of xenografts from control and treated animals; Figure S2: Bioinformatics analyses; Table S1: Celecoxib-controlled gene set (CGS) annotation from Drug Signature DataBase; Table S2: List of primer sequences used for quantitative real-time reverse transcription-PCR analysis; Table S3: List of the antibodies used for WB.

## Abbreviations

ADT	androgen deprivation therapy
AR	androgen receptor
AREG	amphiregulin
BPH	benign prostatic hyperplasia
BIC	bicalutamide
CGS	celecoxib-controlled gene set
COX-2	cyclooxygenase-2
CRPC	castration-resistant prostate cancer
DHT	5-α-dihydrotestosterone
DSigDB	Drug Signature Database
EGF	epidermal growth factor
EGFR-Erb	epidermal growth factor receptor
GO-BP	gene ontology-biological process
GSK3β	glycogen synthase 3β
H&E	hematoxylin and eosin
hnRNP K	heterogeneous nuclear ribonucleoprotein K
mHSPC	metastatic hormone-sensitive PCa
NSAIDs	non-steroidal anti-inflammatory drugs
PARP-1	poly (ADP-ribose) polymerase-1
PCa	prostate cancer
P38	p38MAPK
PPI	protein–protein interaction
