# Supplementary material for: Multifocal Signal Modulation Therapy by Celecoxib: A Strategy for Managing Castration-Resistant Prostate Cancer
Source: Int J Mol Sci. 2019 Dec 3;20(23):6091. doi: 10.3390/ijms20236091 (PMC6929142; doi:10.3390/ijms20236091)
Supplement: Supplementary file 1 [file ijms-20-06091-s001.zip › ijms-635215 supplementary done/supplementary data.pdf]

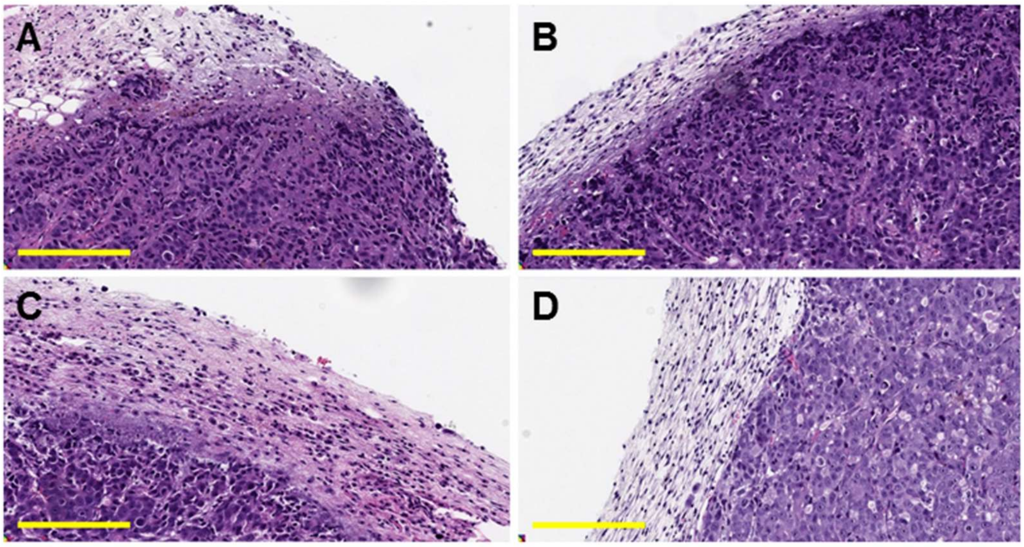

Supplementary Figure S1. Comparison of xenografts from control and treated animals. H&E stained tissues from (A) control, (B) celecoxib, (C) cetuximab, (D) celecoxib and cetuximab treated animals, revealed no histological changes indicating no degeneration or necrosis in all the treated groups. (Light microscope 20x; scale bar represents 200μm).

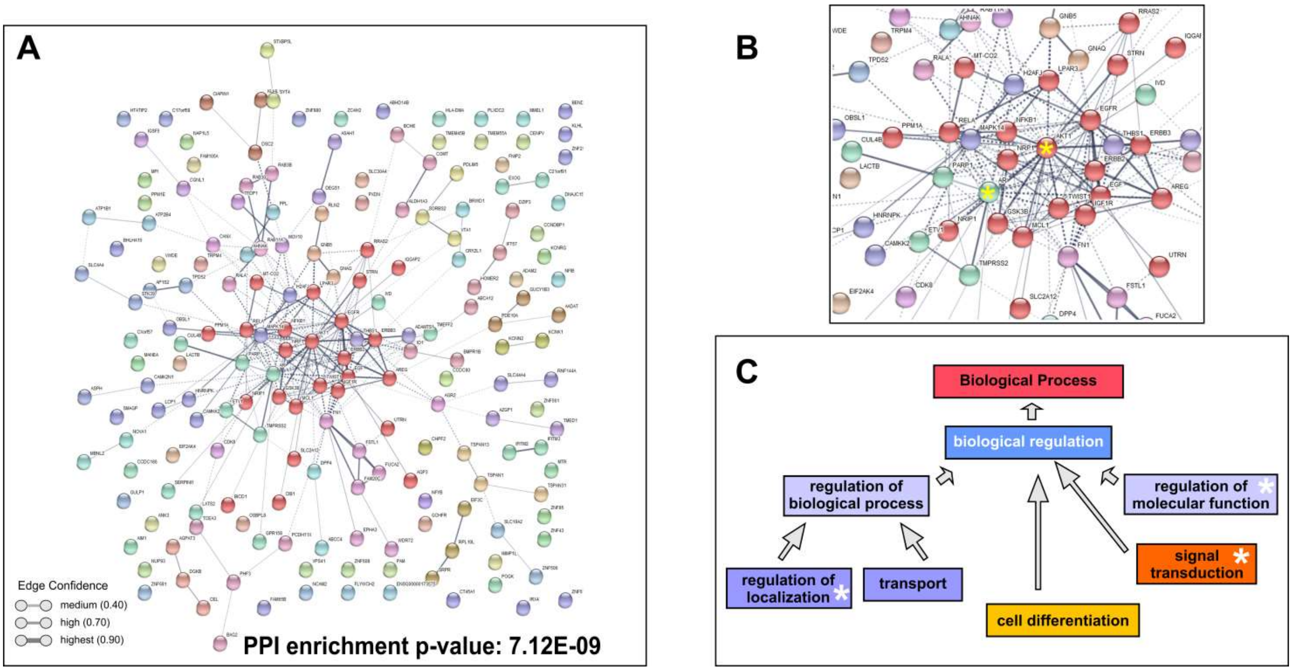

Supplementary Figure S2. Bioinformatics analyses. (A) STRING protein–protein interaction (PPI) network of genes and proteins differentially expressed in PDB and MDB compared to LNCaP and integrated with CGS. Markov Clustering algorithm (inflation parameter 3) was used. Line thickness indicates the strength of data (confidence). (B) Detailed view of *a* showing AR and AKT1, highlighted by asterisks, interactions. (C) Directed Acyclic Graph (DAG) visualization for the top ten highly enriched GO-BP in differentially expressed genes and proteins in PDB, MDB and CGS based on information retrieved from QuickGO. White asterisk, common GO-BP.

Table S2 . List of primer sequences used for quantitative real-time reverse transcription-PCR analysis

Gene name	Primer sequence (5' – 3')
AR	sense TGTCCATCTTGTCGTCTTC
	antisense GAAGCCTCTCCTTCCTCCTG
EGFR	sense ACTGCTGCCACAACCAGTG
	antisense GCCTTCGTCTCGGAATTTG
AREG	sense GCCTTTATGTCTGCTGTGAT
	antisense TCGAAGTTTCTTTCGTTCCTCA
EGF	sense GATTTGTTCTGCTTCCTGATGG
	antisense TCTCTCAAGCACTGAGCCTT
HNRNPK	sense AATCTGATGCTGTGGAATGCTT
	antisense ACCCCAATAATTCCTCCTGCTA
18S	sense ACACGGACAGGATTGACAGATT
	antisense AGACAAATCGCTCCACCAACTA

Table S3 . List of the antibodies used for WB

Antigen	Type	Label	Host	Company	Dilution
Phospho-AKT (Ser473)	Polyclonal		Rabbit	Cell Signaling Technology (CST #9271)	1:1000
AKT	Polyclonal		Rabbit	CST #9272	1:1000
AR	Monoclonal		Mouse	DAKO M3562	1:600
β-actin HPR	Polyclonal		Rabbit	CST #4970	1:4000
EGFR	Polyclonal		Rabbit	CTS #4267	1:1000
ErbB2	Polyclonal		Rabbit	Santa Cruz sc-284	1:1000
ErbB3	Polyclonal		Rabbit	CTS #12708	1:1000
MCL-1	Polyclonal		Rabbit	Bioworld BS1220	1:1000
Cox2	Polyclonal		Rabbit	CTS #12282	1:1000
PARP1	Polyclonal		Rabbit	CST #9542	1:1000
Pospho-GSK-3β (Ser9)	Polyclonal		Rabbit	CTS #9323	1:1000
Pospho –p38 MAPK (Thr180/Tyr182)	Polyclonal		Rabbit	CTS #9211	1:1000
α-Tubulin	Monoclonal		Mouse	SIGMA T6074	1:4000
Mouse Ig G	Polyclonal	Peroxidase	Rabbit	CTS #7076	1:2000
Rabbit Ig G	Polyclonal	Peroxidase	Goat	CTS #7074	1:2000
