# Supplementary material for: Multifocal Signal Modulation Therapy by Celecoxib: A Strategy for Managing Castration-Resistant Prostate Cancer
Source: Int J Mol Sci. 2019 Dec 3;20(23):6091. doi: 10.3390/ijms20236091 (PMC6929142; doi:10.3390/ijms20236091)
Supplement: Supplementary file 1 [file ijms-20-06091-s001.zip › ijms-635215 supplementary done/Table S1.pdf]

Table S1. Celecoxib-controlled gene set (CGS) annotation from Drug SIgnature DataBase. (http://tanlab.ucdenver.edu/DSigDB/DSigDBv1.0/)

Drug	Gene	Type	D4-collection database
celecoxib	AKT1	Text_Mining(decreases(response to substance))	CTD
celecoxib	AKT1	Text_Mining(affects(cotreatment))	CTD
celecoxib	AKT1	Text_Mining(decreases(phosphorylation))	CTD
celecoxib	AKT1	Text_Mining(increases(phosphorylation))	CTD
celecoxib	AKT1	Text_Mining(decreases(reaction))	CTD
celecoxib	AKT1	Text_Mining(increases(activity))	CTD
celecoxib	AKT1	Text_Mining(decreases(activity))	CTD
celecoxib	AKT1	Text_Mining(decreases(expression))	CTD
celecoxib	AR	Potency-Replicate_1=23.9145(uM)	D4 PubChem
celecoxib	AR	Potency-Replicate_1=60.882(uM)	D4 PubChem
celecoxib	AR	Potency-Replicate_1=68.5896(uM)	D4 PubChem
celecoxib	AR	Potency-Replicate_1=23.9145(uM)	D4 PubChem
celecoxib	AR	Potency-Replicate_1=60.882(uM)	D4 PubChem
celecoxib	AR	Potency-Replicate_1=68.5896(uM)	D4 PubChem
celecoxib	AR	Potency-Replicate_1=23.9145(uM)	D4 PubChem
celecoxib	AR	Potency-Replicate_1=60.882(uM)	D4 PubChem
celecoxib	AR	Potency-Replicate_1=68.5896(uM)	D4 PubChem
celecoxib	EGF	Text_Mining(PMID22456425,PMID24528083,PMID23422093,PMID21882253,PMID24817927,PMID22675459,PMID24357184)	BOSS
celecoxib	EGFR	Text_Mining(PMID20848776,PMID21246519,PMID23010081,PMID21217396,PMID24073313,PMID24085777,PMID23749165,PMID23749165,PMID24490043,PMID24490137,PMID21268125,PMID22828609,PMID20585313,PMID20035770,PMID24149137,PMID22252523,PMID22675459)	BOSS

celecoxib	ERBB2	Text_Mining(PMID20103725,PMID20480225,PMID20179229,PMID21384185,PMID23578727)	BOSS
celecoxib	GSK3B	Text_Mining(decreases(phosphorylation))	CTD
celecoxib	GSK3B	Text_Mining(decreases(reaction))	CTD
celecoxib	GSK3B	Text_Mining(increases(expression))	CTD
celecoxib	MAPK14	Text_Mining(affects(cotreatment))	CTD
celecoxib	MAPK14	Text_Mining(increases(activity))	CTD
celecoxib	MCL1	Text_Mining(PMID19665451,PMID21868575,PMID23001726,PMID21345578,PMID23524145,PMID20836993,PMID24637636,PMID24156425,PMID22343223,PMID22504904,PMID24855827,PMID22716247)	BOSS

Legend	
Collection	Description
BOSS	Text mining approach of drug-gene targets using Biomedical Object Search System
CTD	Curation of targhes from Comparative Toxicogenomic Database
TTD	Manual curation targets from the Terapeutics targets Database
D4 PubChem	Drug signature extracted from literatures
D4 ChEMBL	Open Data database containing binding and functional information for drug-like bioactive compounds
PMID	PubMed IDentifier
RED TEXT	Celecoxib-controlled gene set (CGS)
